# Improved classification performance of EEG-fNIRS multimodal brain-computer interface based on multi-domain features and multi-level progressive learning

**DOI:** 10.3389/fnhum.2022.973959

**Published:** 2022-08-04

**Authors:** Lina Qiu, Yongshi Zhong, Zhipeng He, Jiahui Pan

**Affiliations:** School of Software, South China Normal University, Guangzhou, China

**Keywords:** multimodal fusion, electroencephalogram (EEG), mental arithmetic (MA), functional near-infrared spectroscopy (fNIRS), multi-domain features, multi-level learning, motor imagery (MI)

## Abstract

Electroencephalography (EEG) and functional near-infrared spectroscopy (fNIRS) have potentially complementary characteristics that reflect the electrical and hemodynamic characteristics of neural responses, so EEG-fNIRS-based hybrid brain-computer interface (BCI) is the research hotspots in recent years. However, current studies lack a comprehensive systematic approach to properly fuse EEG and fNIRS data and exploit their complementary potential, which is critical for improving BCI performance. To address this issue, this study proposes a novel multimodal fusion framework based on multi-level progressive learning with multi-domain features. The framework consists of a multi-domain feature extraction process for EEG and fNIRS, a feature selection process based on atomic search optimization, and a multi-domain feature fusion process based on multi-level progressive machine learning. The proposed method was validated on EEG-fNIRS-based motor imagery (MI) and mental arithmetic (MA) tasks involving 29 subjects, and the experimental results show that multi-domain features provide better classification performance than single-domain features, and multi-modality provides better classification performance than single-modality. Furthermore, the experimental results and comparison with other methods demonstrated the effectiveness and superiority of the proposed method in EEG and fNIRS information fusion, it can achieve an average classification accuracy of 96.74% in the MI task and 98.42% in the MA task. Our proposed method may provide a general framework for future fusion processing of multimodal brain signals based on EEG-fNIRS.

## Introduction

In recent years, the brain-computer interface (BCI) system has attracted great attention because it can provide another communication channel for people who have lost the ability to move independently by decoding neural signals, and has great application value in the field of rehabilitation (Wolpaw et al., 2002). For example, Han et al. (2019) used an electroencephalogram (EEG)-based BCI system to successfully conduct online binary communication with patients in a fully locked state. The BCI system usually records the user’s brain nerve activity through various brain imaging modes, and then converted into certain instructions, through which users can communicate with the outside world. The brain imaging modalities commonly used in BCI systems include EEG, magnetoencephalography, functional magnetic resonance imaging (fMRI) and functional near-infrared spectroscopy (fNIRS) (Weiskopf et al., 2003; Coyle et al., 2004).

Since each neuroimaging modality has its advantages and disadvantages, combining their complementary characteristics may improve the overall performance of the BCI system (Pfurtscheller et al., 2010; Dähne et al., 2015). Therefore, there is growing interest in hybrid BCI formed by combing two or more modalities. More and more studies have demonstrated that combining different modalities or paradigms can improve the performance of the BCI system, such as hybrid BCI based on P300 and steady-state visually evoked potential(SSVEP) (Wang et al., 2015), hybrid EEG- Electrooculogram (EOG) (Hongtao et al., 2014) and hybrid BCI based on EEG and fNIRS (Naseer and Hong, 2015).

Among various neuroimaging modalities, EEG and fNIRS are widely used in the field of BCI due to their advantages of non-invasiveness, portability, and wide applicability. EEG is a method of recording brain activity using electrophysiological indicators. It reflects the electrophysiological activities of brain nerve cells on the cerebral cortex or scalp surface by recording the changes in electrical waves during brain activity (Olejniczak, 2006). Although EEG is currently one of the most commonly used technology in the field of BCI, it still has some limitations, such as being susceptible to motion artifacts and electrical noise, and having low spatial resolution. fNIRS is an emerging optical brain imaging technology that has attracted much attention in the field of BCI in recent years. It utilizes the good scattering of the main components of blood to 600-900nm near-infrared light, so as to obtain the changes of oxyhemoglobin (HbO) and deoxyhemoglobin (Hb) during brain activity (Matthews et al., 2008; Dashtestani et al., 2019). Compared with EEG, fNIRS is less sensitive to motion artifacts and electrical noise, and has higher spatial resolution (Wilcox and Biondi, 2015) but lower temporal resolution. Due to the complementary properties of EEG and fNIRS, the hybrid BCI based on EEG-fNIRS has attracted more and more attention in recent years (Sinem et al., 2019; Borghea et al., 2020). EEG and fNIRS signals can provide complementary neural activity information to provide a more comprehensive and accurate interpretation of brain function. Numerous studies have shown that EEG-fNIRS hybrid BCI is more likely to improve the performance of traditional single-modal BCI (Koo et al., 2015; Yin et al., 2015; Shin et al., 2017) and has more potential in disease diagnosis based on decoding of patients’ brain activity (Blokland et al., 2014).

In BCI experiments, commonly used task paradigms include motor imaging (MI) and mental arithmetic (MA). MI-based BCI means that there is no actual physical behavior, but the brain’s thoughts are used to imagine the body’s actions, and the controller performs subsequent actual operations. MI is similar to the brain regions activated by actual motor (Holmes and Calmels, 2008; Miltona et al., 2008), which can promote the repair or reconstruction of damaged motor pathways. For example, for limb inconvenience caused by diseases such as paralysis and stroke, the MI-based BCI system can not only help patients control objects, but also can be used as a means of rehabilitation physiotherapy to maximize their recovery of their own athletic ability (Ortner et al., 2012). MA is a task related to memory and cognition in the brain that involves performing arithmetic operations in the brain without the aid of external tools such as pen or paper. Previous studies have demonstrated that the MA task has a positive effect on the fight against degeneration of brain function (Ciftci et al., 2008) and the diagnosis of brain diseases such as schizophrenia (Hugdahl et al., 2001). In Shin et al. (2016) established a public dataset of EEG-fNIRS based on both MI (left vs. right hand motor imagery) and MA(mental arithmetic vs. resting state) tasks, providing an open-access dataset for hybrid BCI. Based on this dataset, various methods have been used to improve the performance of BCI based on MI or MA tasks. For example, Kwon et al. (2020) investigated the feasibility of implementing a compact hBCI system and found that three mental states (mental arithmetic, right-handed motor image, and idle state) could be classified with a classification accuracy of 77.6 ± 12.1% using an hybrid BCI system with only two EEG channels and two fNIRS source-detector pairs. Alhudhaif (2021) combined a k-Means clustering centers difference based attribute weighting method and machine learning algorithm to achieve 99.7% accuracy in MI based dataset and 99.9% accuracy in MA dataset. Nour et al. (2021) proposed a novel classification framework using an optimized convolution neural network (CNN) method for MI tasks to study the contribution of multimodal EEG-fNIRS to BCI performance. Meng et al. (2021) proposed a novel crossing time windows optimization for MA based EEG-fNIRS hybrid BCI, and obtained a classification accuracy of 92.52 ± 5.38%.

The purpose of EEG-fNIRS multimodal systems is to utilize the diversity and complementarity information of EEG and fNIRS signals to maximize the respective advantages of each modality and overcome the limitations of single-modal systems (Li et al., 2019). However, EEG and fNIRS is inherently different, such as sampling rate, temporal and spatial resolution, and noise sensitivity, and the information between EEG and fNIRS signal cannot be communicated. Therefore, how to effectively fuse multimodal brain signals is one of the main challenges for EEG-fNIRS multimodal studies. Currently, there are three commonly used multimodal fusion strategies: data-level fusion, feature-level fusion and decision-level fusion (He et al., 2020). Since data-level fusion directly combines raw and unprocessed data, it is unavoidable that the computational load is too large. The accuracy of feature-level fusion is high, but the existing signal feature-level fusion methods mainly use the feature vector splicing method, which is difficult to eliminate redundant information or enhance key information. Although decision-level fusion is not as accurate as feature-level fusion, it can eliminate redundant information of different modalities to a certain extent. Most of the previous studies on EEG and fNIRS fusion are based on feature-level fusion and achieved promising results. Studies have shown that concatenating features from EEG and fNIRS without any specific feature fusion algorithm can also improve accuracy compared to EEG and fNIRS alone (Buccino et al., 2016). The concurrently recorded EEG and fNIRS features were fused by joint independent component analysis (jICA) can improve the detection rate of mental stress, with detection rates of 91%, 95% and 98% using fNIRS, EEG and fusion of fNIRS and EEG signals, respectively (Al-Shargie et al., 2016). Most recently, the researchers are applying deep learning for integration of features. For example, Sun et al. (2020) used fully connected neural network to design three fusion schemes of linear, tensor and p-th order polynomial to achieve feature fusion of EEG and fNIRS, and found that p-th order polynomial fusion achieved highest classification accuracy for MI task (77.53%) and MA task (90.19%). The above proves that the feature-level fusion strategy can effectively improve the performance of hybrid BCI based on EEG-fNIRS. In addition, some studies have shown that decision-level fusion can also improve the multimodal performance of EEG-fNIRS to a certain extent. For example, Al-Shargie et al. (2017) developed a decision fusion technique to combine the output probabilities of the EEG and fNIRS classifiers based on support vector machine (SVM), and found significant improvement in the detection rate of mental stress by + 7.76% and + 10.57% compared with sole modality of EEG and fNIRS, respectively. Rabbani et al. (Rabbani and Islam, 2021b) proposed a EEG-fNIRS decision fusion model based on long short-term memory network (LSTM) and SVM classifiers, and found that compared to EEG, HbO, and Hb alone, 26 subjects had + 31.83%, + 5.2%, + 15.19% higher detection rates in the decision fusion strategy.

The above studies show that both feature-level fusion and decision-level fusion may effectively fuse EEG and fNIRS information. However, the current multimodal fusion still has the problems of incomplete feature extraction, insufficient multimodal information fusion and poor classification performance. A more comprehensive and systematic approach to efficiently fuse EEG and fNIRS data and fully exploit their complementary potential is needed, which is crucial for improving the performance of EEG-fNIRS-based BCI. Therefore, this paper proposes a novel multimodal fusion framework based on multi-level progressive learning with multi-domain features for fusing EEG and fNIRS information to improve classification performance of EEG-fNIRS multimodal brain-computer interface. The framework mainly includes three processes. The first stage is the multi-domain feature extraction process of EEG and fNIRS in time and frequency domains. The second stage is a feature selection process based on atomic search optimization (ASO). The third stage is a multi-domain feature fusion process based on multi-level progressive machine learning. This study evaluated the effectiveness of the proposed method on MI and MA tasks involving 29 subjects, respectively.

## Materials and methods

### Electroencephalography-functional near-infrared spectroscopy dataset

An open-access hybrid EEG-fNIRS dataset was used in this study (Shin et al., 2016), which consists of two different datasets: (i) dataset A (left- versus right-hand MI) and (ii) dataset B (MA task vs. resting state). In this study, we used both dataset A and dataset B to classify MI- and MA-based brain-computer interface tasks, respectively. Dataset A and dataset B were collected from 29 healthy subjects (14 males), of which 28 were right-handed and 1 was left-handed. Their mean age was 28.5 ± 3.7 years. No subjects reported a history of neurological, psychiatric or another mental disease. Before the experiment, all subjects were informed of the experimental procedures and details, and all signed a written consent form.

**FIGURE 1:**
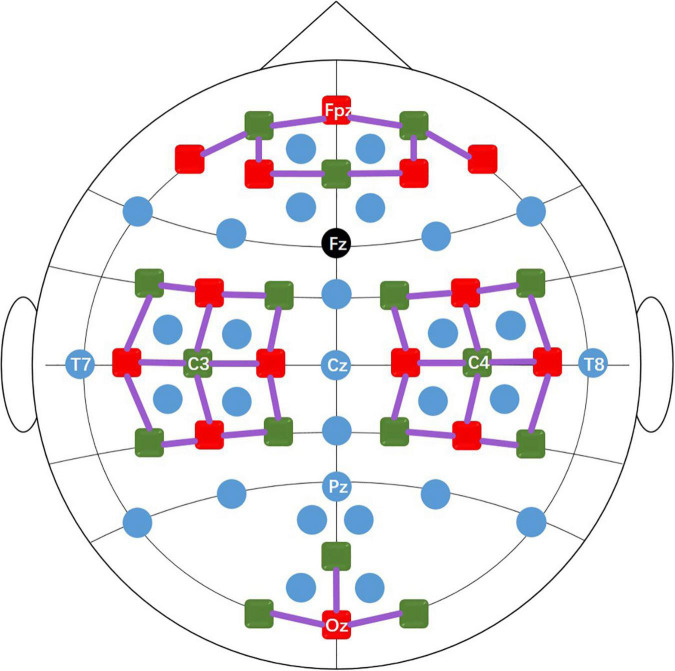
The positions of the EEG electrodes (blue and black dots), fNIRS light sources (red squares), and detectors (green squares). The black dot (Fz) is the ground and the solid purple lines represent the fNIRS channels.

Dataset A and dataset B contain EEG data and fNIRS data. During the experiment, the subject sat in a comfortable armchair in a bright room, 1.6 meters in front of a 50-inch white screen. Subjects were asked to keep their bodies as still as possible during data recording to reduce motion artifacts. EEG data were collected by a multi-channel BrainAmp EEG amplifier (Brain Products GmbH, Gilching, Germany) with 30 active electrodes linked to a mastoids reference at a sampling rate of 1000 Hz. The 30 EEG electrodes were placed on the scalp surface through a customized stretchy fabric cap (EASYCAP GmbH, Herrsching am Ammersee, Germany) according to the international 10-5 system. As shown in Figure 1, the placement positions were AFp1, AFp2, AFF1h, AFF2h, AFF5h, AFF6h, For F3, F4, F7, F8, FCC3h, FCC4h, FCC5h, FCC6h, T7, T8, Cz, CCP3h, CCP4h, CCP5h, CCP6h, Pz, P3, P4, P7, P8, PPO1h, PPO2h, POO1 and POO2, and Fz is the ground electrode. fNIRS data were collected by NIRScout (NIRx GmbH, Berlin, Germany) using two wavelengths of 760 and 850 nm at a sampling rate of 12.5 Hz. 14 sources and 16 detectors (forming 36 channels) were placed on the scalp surface of the frontal area (9 channels around Fp1, Fp2, and Fpz), motor area (12 channels around C3 and C4, respectively) and visual area (3 channels around Oz) of the brain with a source-detector distance of 30mm. The fNIRS optodes were fixed on the same cap as the EEG electrode, and an additional opaque cap over the stretchy fabric cap was used to block ambient light. Figure 1 shows the location of the EEG electrodes and fNIRS optodes. During the data collection process, triggers were sent to EEG and fNIRS instrument at the same time via parallel port using MATLAB to ensure synchronous recording of EEG and fNIRS signals.

**FIGURE 2:**
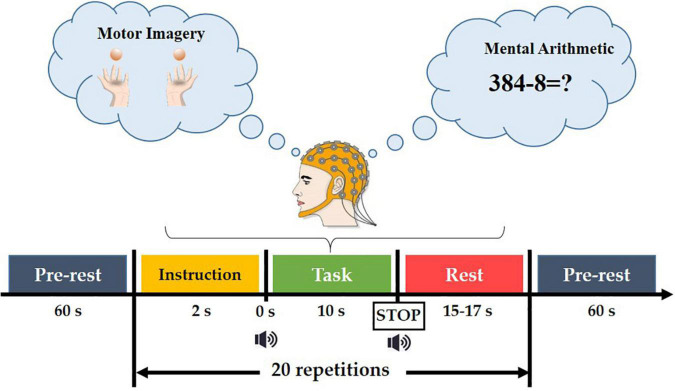
The paradigm of the experiment.

The experimental paradigm consisted of six sessions, in which MI and MA were performed alternately (session 1, 3, 5 for MI tasks and session 2, 4, 6 for MA tasks). As shown in Figure 2, each session consisted of a 60s pre-rest period, 20 repetitions of task process, and a 60-s post-rest period. Each task process started with a 2s visual instruction, followed by a 10s task execution (MI task in Dataset A and MA task in Dataset B), after which was a rest period of 15-17 s.

In the MI task, subjects were taught to execute kinesthetic MI (imagining their hands opening and closing as they grabbed a ball). A black arrow pointing to the left or right appeared for 2 seconds in the center of the screen for the visual instruction. During the task period, the arrow vanished with a short beep sound, and a black fixation cross emerged. The participants were asked to envision holding their hands (opening and closing them) at a 1 Hz rate. Data for MI tasks were collected in Database A.

In MA tasks, the 2-s task visual instruction, that is, an instruction using an initial subtraction, for example “three digits minus one digit” (such as 384-8) was displayed on the screen for 2 s for the preparation of the given task. At the same time, the subjects were asked to remember the numbers. Before the initial subtraction disappeared, a short beep sound was played, prompting the subjects to perform a 10-s MI or MA task, that is, subtracting a single-digit number from the result of the previous subtraction. Each task execution ended with a short beep and a ‘stop’ displayed on the screen for 1 s. After the task was executed, there was a 15-17 second rest. This task process was repeated 20 times in a single session. More details can be found in reference (Shin et al., 2016). Data for MA tasks were collected in Database B.

The MI and MA datasets are publicly available which can be found in the following link: http://doc.ml.tu-berlin.de/hBCI.

### Data preprocessing

All data preprocessing was performed using MATLAB R2020a (MathWorks, Natick, MA, United States) and bbci toolbox (Blankertz et al., 2016). For the preprocessing of EEG data, the raw EEG data was first re-referenced using the common average reference and filtered with a passband of 0.5–50 Hz using a fourth-order Chebyshev type II filter. Most of the previous studies based on EEG signals also used this filter in preprocessing (Goncharova et al., 2003; Fatourechi et al., 2007; Laghari et al., 2014). Then, the EOG rejection based on independent component analysis was performed by automatic artifact rejection toolbox in EEGLAB. After removing the EOG artifacts, the EEG signals of all channels were down-sampled to 200 Hz, and the data of each task process (i.e., 2s visual instruction + 10s task execution + 15-17 s rest) was extracted, and a total of 60 (3 sessions x 20 repetitions) task process data. For the preprocessing of fNIRS data, the raw fNIRS data was first down-sampled to 10 Hz, and then the concentration changes of HbO and Hb were first converted from raw optical data by applying the modified Beer–Lambert law (Kocsis et al., 2006). The HbO and Hb data were band-pass filtered at 0.01-0.09 Hz (a 3rd order zero-phase Butterworth filter) to remove such as from heartbeat (∼1 Hz), respiration-induced venous pressure waves (∼0.2 Hz), arterial pressure waves (∼0.2 Hz) and arterial pressure oscillations (Mayer waves∼0.1 Hz) artifacts. Then, like the EEG data processing, 60 task process data (i.e., 2s visual instruction + 10s task execution + 15-17 s rest) were extracted, and the baseline correction was performed for each task process data by subtracting the average value of the 2s visual instruction period. All channels of EEG and fNIRS were used for further data processing. For EEG and fNIRS data, we set the 1-second data as a subtask, that is, each task contains 10 subtasks, and each session contains a total of 20 (repetitions) x 10 (subtasks) = 200 subtasks. Therefore, each subject had a total of 3(sessions)*200 (subtasks) = 600 subtasks in an MI or MA task.

### Proposed multimodal fusion framework

**FIGURE 3:**
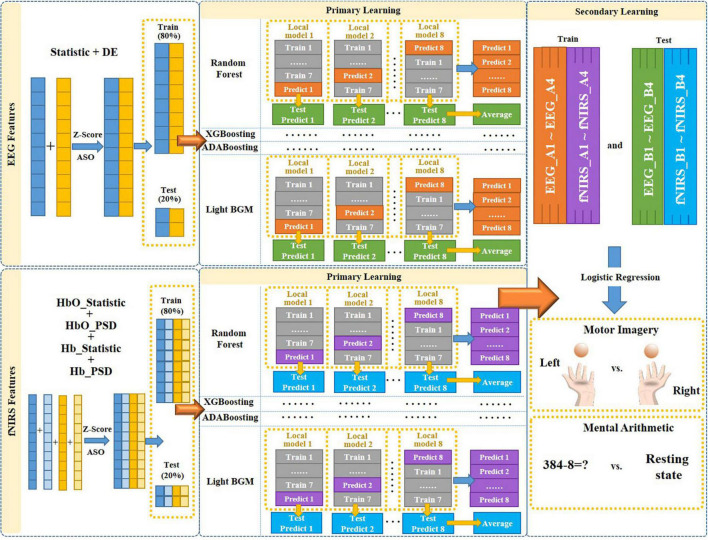
The overall architecture of the proposed multimodal fusion framework.

To efficiently fuse EEG and fNIRS data and fully exploit their complementary potential is needed, which is crucial for improving the performance of EEG-fNIRS-based BCI. In this study, we proposed a novel multimodal fusion framework based on multi-level progressive learning with multi-domain features to improve classification performance of EEG-fNIRS multimodal brain-computer interface. As shown in Figure 3, the framework mainly includes three processes. The first stage is the multi-domain feature extraction process of EEG and fNIRS in time and frequency domains. The second stage is a feature selection process based on Z-Score and ASO algorithm. The third stage is a multimodal feature fusion process based on multi-level progressive machine learning.

### Feature extraction

In order to make full use of brain signals, we extracted time-domain and frequency-domain features from the preprocessed EEG data and fNIRS data, respectively, for the classification of MI tasks (left-hand vs. right-hand) and classification of MA tasks (MA vs. resting state).

#### Time domain feature

For time domain features, we extracted six statistic features of EEG and fNIRS, including signal mean, signal variance, mean of the first difference absolute value, mean of the normalized first difference absolute value, mean of the second difference absolute value and mean of the normalized second difference absolute value. The formulas for calculating these statistic features are shown as (Qiu et al., 2022).

#### Frequency domain feature

For the frequency domain features of EEG signals, we extracted the differential entropy (DE) feature in the range of 0.5–45 Hz using short-term Fourier transform, which is calculated as follows:


(1) $$\mathrm{DE}=h\,\left(x\right)-\int_{-\infty }^{+\infty }\frac{1}{\sqrt{2\,\pi \,\sigma^{2}}}\,e^{-\frac{{\left(x-\mu \right)}^{2}}{2\,\sigma^{2}}}\,\log\left(\frac{1}{\sqrt{2\,\pi \,\sigma^{2}}}\,e^{-\frac{{\left(x-\mu \right)}^{2}}{2\,\sigma^{2}}}\right)$$

$$\mathrm{dx}=\frac{\log\left(2\,\pi \,e\,\sigma^{2}\right)}{2}$$

where μ and σ represent the mean and standard deviation of the signal, respectively, and *e* is the Euler constant.

For the frequency domain feature of fNIRS signals, we extracted the power spectral density (PSD) feature of HbO and Hb in the range of 0.01–0.1 Hz using Fourier transform, respectively. The PSD feature, like the DE feature, is based on the Fourier transform. It converted the fluctuations of the signal surface topography into the spatial frequency domain within the range of the specified frequency domain, so as to obtain the intensity spectral line distribution of the high, medium and low frequency energy topography components. The specific process is as follows: For a discontinuous HbO and Hb signal *x*(*n*) with limited length, the discrete Fourier transform is firstly performed, and the formula is:


(2) $$X_{N}\,\left(e^{-j\,\omega }\right)=\sum_{n=1}^{N}x\,\left(n\right)\,e^{-j\,\omega \,n}$$

(3) $$P\,\left(\omega \right)=\frac{1}{N}\,{\left|X_{N}\,\left(e^{-j\,\omega }\right)\right|}^{2}=\frac{1}{N}\,{\left|\sum_{n=1}^{N}x\,\left(n\right)\,\left(e^{-j\,\omega }\right)\right|}^{2},n=1,\ldots ,N$$

where *X*_*N*_(*e*^−*j*ω^) is the Fourier transform of the sequence *x*(*n*) and *P*(ω) is the final result of the average periodogram estimated power spectrum.

### Feature selection

Time-domain feature and frequency-domain feature are features from two different dimensions. When multiple different types of features are used, simple feature splicing often leads to redundant information, making it is difficult to fully utilize the effective information of multiple features. To combine the time and frequency domain features from EEG or fNIRS more efficiently, we utilized the Atom Search Optimization (ASO) algorithm for feature selection on the combined time and frequency domain features in this study. Specifically, we first converted all features into the same dimension using Z-Scores (Aris et al., 2018), and then used the ASO algorithm for feature selection. The ASO algorithm was proposed by Zhao et al. (2018) in 2018, which is inspired by basic molecular dynamics. All matter in nature is composed of atoms. Atoms have mass and volume. In an atomic system, all atoms interact and are in a constant state of motion. The role is very complex. With the development of science and technology, molecular dynamics has developed rapidly in recent years, and it is already possible to use computers to simulate the laws of physical motion of atoms and molecules. The ASO algorithm mainly simulates the displacement of atoms caused by the interaction of the interaction force generated by the Lennard-Jones potential and the binding force generated by the bond length potential in the atomic motion model, and uses attraction and repulsion to realize global search and local search. In ASO, the position of each atom in the search space represents a solution, and this solution is related to the atomic mass. A better solution means atoms with heavier mass and vice versa. All atoms in the group attract or repel each other depending on their distance, causing the lighter atoms to move toward the heavier ones. Heavier atoms have less acceleration, which makes them seek better solutions in local space. Lighter atoms have greater acceleration, which allows them to search for new regions of the global search space where better solutions may exist. Previous studies have demonstrated that this algorithm can be used to remove redundant information between features in different frequency domains, leaving complementary and useful information (Ghosh et al., 2020).

### EEG-fNIRS fusion

Determining the trustworthiness of each mode to the objective, then coordinating and making joint judgments, is what decision-level fusion entails. In the decision-level fusion, each modal feature is classified independently at the decision level to generate local judgments based on individual features, which are then integrated by decision-level fusion methods. In this paper, we proposed a progressive learning method to achieve the decision-level fusion of different features between two different modalities, so as to solve the problem of information incompatibility between different modalities. Progressive learning is one of the algorithms in ensemble learning, which is an algorithm that is learned by combining multiple individual machine learning algorithms. Progressive learning method contains a total of two-level learning process, primary learning and secondary learning. We selected four machine learning models, namely Random Forest, eXtreme gradient boosting (XGBoosting), adaptive boosting (ADABoosting) and light gradient boosting machine (LGBM), as the primary learning models {*λ_1_*, *λ_2_*, *λ_3_*, *λ_4_*}, and Logistic Regression as the secondary learning model λ.

The proposed multimodal fusion framework based on progressive learning is shown in Figure 3. First, we combined the time-domain and frequency-domain features of a single modality (EEG or fNIRS), respectively, to form a new feature matrix. Then, the ASO algorithm was used to perform feature selection on the new feature matrix to remove the information redundancy caused by multi-domain features. Next, the selected multi-domain features of EEG and fNIRS were used as the input for multi-level progressive learning, respectively. In the multi-level learning process, we used a total of four classification models (Random Forest, XGBoosting, ADABoosting, and LGBM) as the training model for the primary learning stage, and Logistic Regression as the training model for the secondary learning.

In the primary learning stage, we first divided the multi-domain features of a single modality (EEG or fNIRS) into two parts in an 8:2 ratio as the input to the four learning models. Those 80% of the features were further divided into 8 small parts {*k*_1_, *k*_2_, …, *k*_8_}, and one of the small parts *k*_*i*_ (*i* = 1.8) was selected as the test set in turn, and the remaining as the training set. Each training and testing produce a local model and a set of predicted values, namely “Local model 1∼Local model 8” and “Predict 1∼Predict 8” in Figure 3. Then, each local model was used to predict the corresponding 20% unimodal features (those obtained after feature selection), and a total of 8 test prediction values were obtained, namely “Test Predict 1∼Test Predict 8” in Figure 3. Their average was recorded as “Average.” Therefore, after the primary learning with four learning models, each single modality (EEG or fNIRS) corresponds to 4 sets of “Predict 1∼Predict 8” (each set was named as Ai, *i* = 1.4) and 4 sets of “Average” of Test Predict values (each set named as Bi, *i* = 1.4). That is, {EEG_A1, …, EEG_A4} and {EEG_B1, …., EEG_B4} for EEG and {fNIRS_A1,…, fNIRS_A4} and {fNIRS_B1, …, fNIRS_B4} for fNIRS.

In the secondary learning stage, {EEG_A1, …, EEG_A4, fNIRS_A1, …, fNIRS_A4} was used as training set, {EEG_B1, …, EEG_B4, fNIRS_B1, …, fNIRS_B4} was used as test set, and Logistic Regression was used as classification model to obtain final classification results for MI and MA tasks.

This paper proposed multimodal fusion framework based on multi-level progressive learning with multi-domain features to improve the classification performance of the EEG-fNIRS multimodal brain-computer interface. The framework mainly includes three processes: (1) Multi-domain feature extraction process of EEG and fNIRS in time and frequency domains. (2) Feature selection process based on ASO algorithm. (3) Multimodal feature fusion process based on multi-level progressive machine learning. This study evaluates the effectiveness of the proposed method on MI and MA tasks involving 29 subjects. Our proposed framework is outlined in the following pseudocode (Algorithm 1).

**ALGORITHM 1:** Training and optimization procedures of EEG-fNIRS fusion based on our proposed framework.

**Inputs:** time domain features of EEG *E1* = {(*E1x*_*m*_, y_*m*_)}; frequency domain features of EEG *E2* = {(*E2x*_*m*_, y_*m*_)}; time domain features of fNIRS *F1* = {(*F1x*_*m*_, y_*m*_)}; frequency domain features of fNIRS *F2* = {(*F2x*_*m*_, y_*m*_)}; number of primary learning models *T*; primary learning models λ_1_, λ_2_, λ_3_, λ_4_; secondary learning model λ
**Outputs:** trained model *M* based on our proposed method; predicted labels *L*;
1. **set** *E* = *E1 ∪ E2* = {(*Ex*_*m*_, *y*_*m*_)},
2. *F* = *F1 ∪ F2* = {(*Fx*_*m*_, *y*_*m*_)}% Combine the time and frequency domain features
3. *D*_1_ = *E*’, *D*_2_ = *F*’% feature selection using ASO algorithm for *E* and *F*:
4. **for** *t* = 1, …, *T*:
5. *h*_*t*1_ = λ_*t*_ (*D*_1_)% Train a primary individual learner *h*_*t*1_ and *h*_*t*2_ by applying the primary
6. *h*_*t*2_ = λ_*t*_ (*D*_2_)% learning models
7. **end for**
8. *D*’ = ∅; % Generate a new data set
9. **for** *i* = 1,…,k:% k-flod cross-validation
10. **for** *t* = 1,…,*T*:
11. *h*_*t*1_’ = *h*_*t*1_ (*E1x*_*i*_), *h*_*t*2_’ = *h*_*t*2_ (*F1x*_*i*_),
12. *h*_*t*_ = *h*_*t*1_’ ∪ *h*_*t*2_’
13. *Zit* = *h*_*t*_ (*x*_*i*_)
14. **end for**
15. *D*’ = *D*’ ∪ ((*Z*_*i*1_, *Z*_*i*2_, …, *Z*_*iT*_), *y*_*i*_)
16. **end for**
17. *h*’ = λ (*D*’);% Train the secondary learner *h*’ by applying the secondary learning
18. % algorithm λ to the new data set *D*’
19. *M* (*x*) = *h*’ (*h*_1_ (*x*), *h*_1_ (*x*),…, *h*_*T*_ (*x*))
20. **return** predicted labels *L* and trained model *M* based on our proposed method

## Results

This section mainly presents the classification accuracy of EEG and fNIRS-based single-domain and multi-domain features, single- and multi-modality, the traditional fusion method and the proposed fusion method on MI and MA tasks.

**FIGURE 4:**
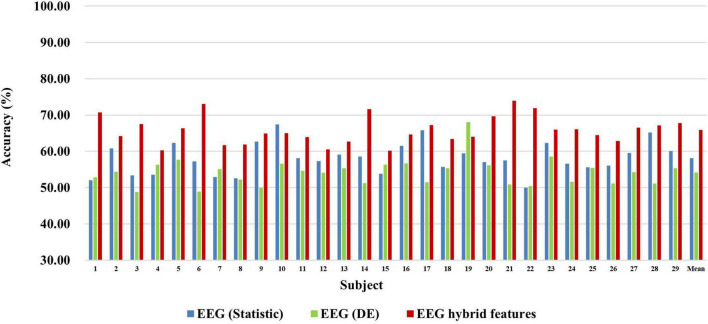
Classification accuracies of EEG-based statistic features and DE feature and their multi-domain hybrid features in the MI task for 29 subjects. The abscissa represents the test number, and the ordinate represents the classification accuracy.

**FIGURE 5:**
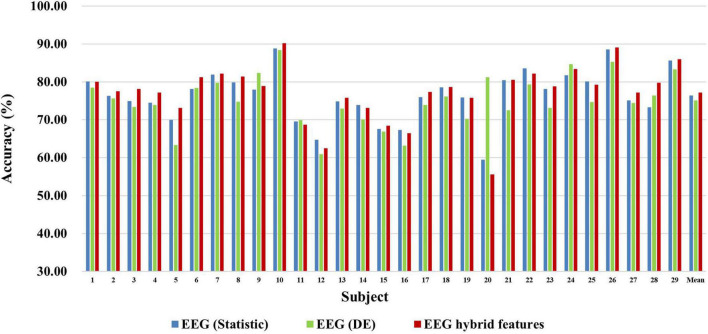
Classification accuracies of EEG-based statistic feature and DE feature and their multi-domain hybrid features in the MA task for 29 subjects. The abscissa represents the test number, and the ordinate represents the classification accuracy.

First, we explored the classification accuracy of MI and MA tasks based on the time- and frequency-domain features of EEG and fNIRS signals. The time-domain features of EEG and fNIRS used here include a variety of timing statistical features, and the frequency-domain features included DE feature of EEG and PSD feature of fNIRS. These time-domain and frequency-domain features were normalized by Z-Score and then feature selection using ASO algorithm before being used for classification. In the classification of both MI and MA tasks, a Random Forest classifier was used to classify time-domain features, frequency-domain features, and time-frequency hybrid features from EEG and fNIRS as input, respectively. Figures 4, 5 show the classification accuracy of EEG-based statistic feature and DE feature and their multi-domain hybrid features in MI and MA tasks, respectively, for 29 subjects. As shown in Figures 4, 5, in the classification of MI and MA tasks, all subjects show higher classification accuracy in the EEG-based time-frequency domain hybrid features than single time or frequency domain features. In the EEG-based MI task classification, the average classification accuracies of 29 subjects based on statistic feature, DE feature and their multi-domain hybrid features were 58.01 ± 4.33%, 54.17 ± 3.79%, and 65.87 ± 3.78%, respectively. In the EEG-based MA task classification, the average classification accuracies of 29 subjects based on statistic feature, DE feature and their multi-domain hybrid features of EEG were 76.42 ± 6.73%, 75.06 ± 6.68%, and 80.75 ± 7.60%, respectively.

**FIGURE 6:**
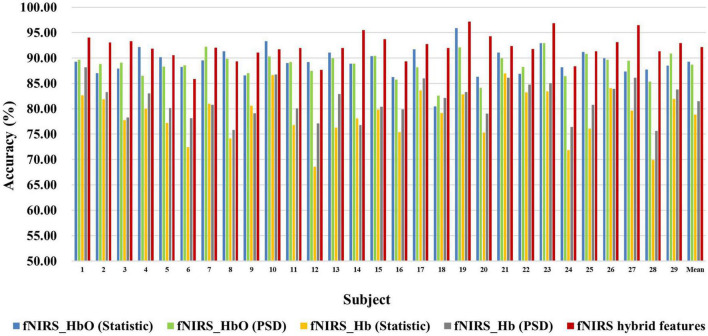
Classification accuracies of fNIRS-based statistic features and PSD features of HbO and Hb and their multi-domain hybrid features in the MI task for 29 subjects. The abscissa represents the test number, and the ordinate represents the classification accuracy.

**FIGURE 7:**
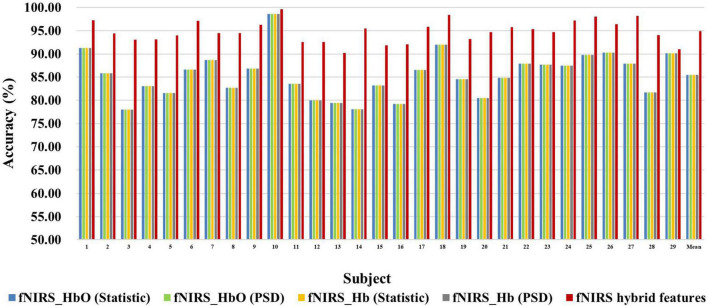
Classification accuracies of fNIRS-based statistic features and PSD features of HbO and Hb and their multi-domain hybrid features in the MA task for 29 subjects. The abscissa represents the test number, and the ordinate represents the classification accuracy.

Figures 6, 7 show the classification accuracy of fNIRS-based statistic feature and PSD feature and their multi-domain hybrid features in MI and MA tasks, respectively, for 29 subjects. It can be seen that the classification accuracy of both the MI and MA tasks based on the time-frequency domain hybrid features of fNIRS for all subjects is higher than that of the single time domain or frequency domain features. In the fNIRS-based MI task classification, the average classification accuracies of 29 subjects based on the statistic feature of HbO, the PSD feature of HbO, the statistic feature of Hb, the PSD feature of Hb, and their multi-domain hybrid features were 89.26 ± 2.87%, 78.87 ± 4.71%, 88.71 ± 2.37%, 81.51 ± 3.57%, and 92.19 ± 2.59%, respectively. In the fNIRS-based MA task classification, the average classification accuracies of 29 subjects based on the statistic feature of HbO, the PSD feature of HbO, the statistic feature of Hb, the PSD feature of Hb, and their multi-domain hybrid features were 92.51 ± 2.27%, 85.74 ± 5.20%, 90.00 ± 1.99%, 92.85 ± 2.23%, and 94.88 ± 2.35%, respectively. From the classification results of MI and MA based on EEG and fNIRS features, it can be seen that the classification accuracy can be effectively improved by combining time domain and frequency domain features. Among them, the classification accuracies of MI and MA tasks based on EEG time-frequency multi-domain features is 7.86 and 4.33% higher than that of EEG time-domain single-domain features, and 11.70% and 5.69% higher than EEG frequency-domain single-domain features, respectively. The classification accuracies of MI and MA tasks based on fNIRS time-frequency multi-domain features is 3.48% and 4.88% higher than fNIRS time-domain single-domain features, respectively, and 13.32% and 9.14% higher than fNIRS frequency-domain single-domain features, respectively.

Previous studies have shown that a multimodal combination based on EEG and fNIRS can provide better performance than single modality (He et al., 2020). But there is currently a lack of a systematic approach to properly fuse EEG-fNIRS data and exploit their complementary potential. Therefore, we proposed a fusion method based on progressive multi-level learning to deeply fuse different features between two different modalities, EEG and fNIRS, to classify MI and MA tasks more efficiently. The fusion method mainly includes two levels of learning: primary learning and secondary learning. In the primary learning, we used Random Forest, XG Boosting, ADA Boosting and Light GBM four machine learning models for a single modality to learn multi-domain features from EEG and fNIRS, respectively. In secondary learning, we further used Logistic Regression for final classification based on primary learning results. To verify the effectiveness of the proposed fusion method, we compared it with the direct splicing method. The direct splicing method here refers to simple matrix splicing of the extracted EEG features (including statistical features and DE features) and fNIRS features (including statistical features and PSD features of HbO and Hb) without any other processing, and then using the random forest algorithm for classification. The classification results based on direct splicing were 8-fold cross-validated. Table 1 shows the classification accuracies on MI and MA tasks obtained by fusing EEG and fNIRS multimodal features based on the proposed fusion method and the direct splicing method, respectively, for 29 subjects. It can be observed that in both the MI and MA tasks, the accuracy obtained by each subject in the fusion of EEG and fNIRS multimodal features based on our proposed fusion method is higher than the direct splicing method. The average classification accuracies of 29 subjects for MI and MA tasks based on the direct splicing method was 80.47 ± 4.68% and 85.43 ± 4.79%, respectively, while the average classification accuracy based on our proposed fusion method was 96.74 ± 1.96% and 98.42 ± 1.52%, respectively. In the classification of MI tasks, the fusion method of direct splicing improves the accuracy by 14.60% compared with the EEG-based single modality, and reduces the accuracy by 11.72% compared with the fNIRS-based single modality. In the classification of the MA task, the fusion method of direct splicing improves the accuracy by 4.68% compared with the EEG-based single-modality, and reduces the accuracy by 9.45% compared with the fNIRS-based single-modality. That is, the multimodal fusion method of direct splicing plays the role of ‘positive fusion’ compared with the single-modality based on EEG, but plays the role of “negative fusion” when compared with the single-modality based on fNIRS. The emergence of the phenomenon of “negative fusion” indicates that the features of EEG and fNIRS have not been effectively fused. The reason may be that the features of EEG and fNIRS are cross-modal, and their essence, acquisition mechanism and dimensions are different.

**TABLE 1:** The classification accuracies on MI and MA tasks obtained by fusing EEG and fNIRS multimodal features based on the proposed fusion method and the direct splicing method for 29 subjects.

Task	MI		MA	
		
Features Subject	EEG-fNIRS (Direct splicing)	EEG-fNIRS (Proposed fusion method)	EEG-fNIRS (Direct splicing)	EEG-fNIRS (Proposed fusion method)
1	81.49%	95.03%	91.26%	100.00%
2	80.69%	97.11%	85.78%	99.54%
3	79.69%	96.99%	77.97%	94.94%
4	83.59%	97.21%	83.03%	98.31%
5	80.79%	96.18%	81.54%	96.51%
6	74.25%	97.73%	86.62%	99.48%
7	84.63%	98.23%	88.64%	97.76%
8	71.39%	96.35%	82.69%	98.32%
9	82.93%	99.01%	86.78%	98.39%
10	86.13%	98.89%	98.63%	98.31%
11	77.23%	97.13%	83.52%	97.61%
12	75.90%	97.37%	79.96%	93.47%
13	76.45%	98.23%	79.44%	96.74%
14	77.97%	98.36%	78.07%	99.68%
15	85.30%	95.08%	83.16%	97.12%
16	74.96%	96.37%	79.17%	99.77%
17	83.60%	97.93%	86.56%	98.88%
18	77.33%	96.44%	91.97%	99.80%
19	81.49%	98.73%	84.56%	98.84%
20	74.51%	98.33%	80.45%	99.23%
21	89.53%	94.10%	84.84%	98.97%
22	85.54%	95.64%	87.88%	98.58%
23	84.81%	98.34%	87.64%	99.73%
24	77.19%	94.51%	87.42%	99.22%
25	82.85%	90.13%	89.76%	99.85%
26	83.65%	97.42%	90.27%	98.54%
27	83.87%	97.24%	87.90%	99.21%
28	71.46%	98.06%	81.72%	97.68%
29	84.48%	93.18%	90.14%	99.75%
Mean	80.47%	96.74%	85.43%	98.42%
Standard deviation	4.68%	1.96%	4.79%	1.52%

However, after the fusion of our proposed method, the classification accuracy of EEG-fNIRS is as high as 96.74 ± 1.96% in the MI task, and the classification accuracy in the MA task is as high as 98.42 ± 1.52%. It can be observed that the proposed fusion method improves the accuracy by 30.87% over the EEG-based single modality in the MI task and 4.55% over the fNIRS-based single modality. It achieves a 17.67% improvement over the EEG-based single-modality accuracy in the MA task and a 3.54% improvement over the fNIRS-based single-modality accuracy. Moreover, compared with the EEG-fNIRS fusion method based on simple splicing, the classification accuracy of our proposed method in the MI task is improved by 16.27% and the classification accuracy in the MI task is improved by 12.99%. Obviously, our proposed fusion method can effectively fuse the multimodal features of EEG and fNIRS, thereby improving the classification accuracy of BCI based on MI and MA tasks.

## Discussion

In recent years, multimodal hybrid BCI has attracted more and more attention. Many studies have shown that using the complementarity between different modalities of brain imaging techniques can obtain richer brain information and eliminate redundant information, thereby improving the performance of BCI. However, due to the different acquisition mechanisms, temporal and spatial resolutions, and noise susceptibility of different modal technologies, the information between various modalities may not be interoperable. How to use the diverse and complementary information of different modalities to maximize their respective advantages and overcome the limitations of a single-modal system is one of the main challenges in the application of multimodal BCI. To address this issue and improve the performance of BCI based on EEG-fNIRS, in this study, we proposed a novel multimodal fusion method based on multi-level progressive learning with multi-domain features. The proposed method was evaluated using a public dataset based on EEG-fNIRS, which contains two tasks, MI (left-hand and right-hand) and MA (MA and resting state).

Currently the classification framework based on MI and MA tasks generally concentrates on the pattern of classifier with single-domain feature, which can only provide limited information useful for final classification, thus may lead to unsatisfactory performance. Therefore, in this study, we extracted the temporal and spectral feature information of EEG and fNIRS-based brain activity from multiple domains (time and frequency domains), which can mine the different and complementary information for MI and MA pattern classification. There are currently few studies on the classification of MI tasks and MA tasks (especially MA tasks) based on multi-domain hybrid features of EEG or fNIRS. From the classification results of single-domain and multi-domain features based on EEG and fNIRS (Tables 1, 2), it can be seen that combining time-domain and frequency-domain features can effectively improve the classification accuracies of MI and MA tasks. In the MI task, the classification accuracy of EEG-based multi-domain features is up to 11.70% higher than that of single-domain, and the classification accuracy of fNIRS-based multi-domain features is up to 13.32% higher than that of single-domain. In the MA task, the classification accuracy of multi-domain features based on EEG is up to 5.69% higher than that of single domain, and the classification accuracy of multi-domain features based on fNIRS is up to 9.14% higher than that of single domain. This conclusion is consistent with previous EEG-fNIRS-based studies finding that using multi-domain features can improve BCI classification performance based on MI and MA tasks. Table 2 lists the classification accuracy of MI and MA tasks based on single-domain features and multi-domain hybrid features based on EEG or fNIRS. As can be seen from Table 2, the conclusions of the previous related studies and our study jointly illustrate that multi-domain features can provide higher BCI classification performance than single-domain features.

**TABLE 2:** Classification accuracies of MI and MA tasks based on single-domain features and multi-domain hybrid features based on EEG or fNIRS.

Studies	Tasks	Classification Algorithms	Modalities	Features	Accuracies
Shin et al., 2016	MI	Linear Discriminant Analysis (LDA)	EEG	Power Spectrum	73.10%
				Common Spatial Pattern	63.39%
				Hybrid Features	73.28%
			fNIRS	Mean Value of HbO Channel Wise	82.76%
				Mean Value of Hb Channel Wise	79.66%
				Modified Common Spatial Pattern	78.74%
				Hybrid Features	86.84%
	MA		EEG	Power Spectrum	82.64%
				Common Spatial Pattern	77.24%
				Hybrid Features	84.6%
			fNIRS	Mean Value of HbO Channel Wise	82.76%
				Mean Value of Hb Channel Wise	79.66%
				Modified Common Spatial Pattern	78.74%
				Hybrid Features	86.84%
Li et al., 2021	MI	Deep Forest	EEG	Time-frequency	62.00%
				Common Spatial Pattern	72.00%
				Fusion	75.00%
Saadati et al., 2020	MI	Deep Neural Networks (DNN)	fNIRS	HbO_Mean	70.00%
				Hb_Mean	-
				Hybrid Features	80.00%
Yin et al., 2015	MI	Extreme Learning Machines	EEG	Power	70.00%
				Instantaneous Amplitude	72.00%
				Instantaneous Phase	81.00%
				Instantaneous Frequency	79.00%
				Hybrid Features	88.00%
			fNIRS	HbO_Mean	70.00%
				Hb_Mean	72.00%
				Total HbO and Hb	81.00%
				Differences between HbO and Hb	79.00%
				Hybrid Features	88.00%
Zhang et al., 2021	MI	LDA	EEG	Power Spectral Density	74.00%
				Common Spatial Pattern	75.90%
				Wavelet Transform	83.20%
				Hybrid Features	84.70%
**Our work**	MI	Random Forest	EEG	Statistic	58.01 ± 4.33%
				DE	54.17 ± 3.79%
				Hybrid Features	65.87 ± 3.78%
			fNIRS	HbO_statistic	89.26 ± 2.87%
				HbO_PSD	85.74 ± 5.20%
				Hb_statistic	90.00 ± 1.99%
				Hb_PSD	81.51 ± 3.57%
				Hybrid Features	92.19 ± 2.95%
	MA		EEG	Statisitc	76.42 ± 6.73%
				DE	75.06 ± 6.68%
				Hybrid Features	80.75 ± 7.60%
			fNIRS	HbO_statistic	92.51 ± 2.27%
				HbO_PSD	85.74 ± 5.20%
				Hb_statistic	90.00 ± 1.99%
				Hb_PSD	92.85 ± 2.23%
				Hybrid Features	94.88 ± 2.35%

Determining a systematic approach to properly fuse EEG-fNIRS data and exploit their complementary potential is critical for improving the performance of EEG-fNIRS-based BCI. Incorrect fusion methods such as direct feature splicing not only cannot improve performance, but may lead to the appearance of ‘negative fusion’ and reduce performance. In this study, we proposed a novel multimodal fusion method based on multi-level progressive learning with multi-domain features, which deeply fused multi-domain features of two modalities, EEG and fNIRS, at the decision-level. Our fusion results show that, the classification accuracy based on EEG-fNIRS multi-domain features is as high as 96.74 ± 1.96% in the MI task, and the classification accuracy in the MA task is as high as 98.42 ± 1.52%. These results are greatly improved over both single modality and traditional fusion methods of direct feature splicing. It can be seen that our proposed fusion method can effectively fuse the multimodal features of EEG and fNIRS. Table 3 presents the classification accuracies of the fused EEG-fNIRS multi-modality for MI and MA tasks on the same open dataset. At present, most of the studies based on the same dataset (Shin et al., 2016) fused EEG and fNIRS at the feature-level (Shin et al., 2016; Meng et al., 2021; Nour et al., 2021), and some perform dual fusion at the feature-level and decision-level (Rabbani and Islam, 2021a). It can be seen from Table 3 that our fusion results in MI and MA tasks are better than most related studies (Shin et al., 2016; Meng et al., 2021; Rabbani and Islam, 2021a), that is, the classification accuracies were higher. Nour et al. (2021) proposed a novel multi-bandwidth classification framework based on an optimized convolutional neural network (CNN) to fuse EEG and fNIRS, and achieved 99.85% classification accuracy on the MA task. Although the fusion result of this study is slightly higher than our fusion result of 98.42%, their fusion method was based on deep learning, which requires a large amount of computation, and the fusion model was only verified on a single MA task. However, our fusion framework mainly relies on machine learning and achieves good classification accuracy on both MI and MA tasks. All in all, our proposed fusion framework is more competitive with existing fusion methods.

**TABLE 3:** The classification accuracies of the fused EEG-fNIRS multi-modality for MI and MA tasks on the same open dataset.

Studies	Tasks	Classification algorithms	Multi-modality	Fusion strategies	Accuracies
Shin et al., 2016	MI	LDA	EEG-fNIRS	Feature-level	75.9%
	MA	LDA	EEG-fNIRS	Feature-level	86.2%
Rabbani and Islam, 2021a	MI	SVM + LDA	EEG-fNIRS	Feature-level + Decision-level	78.56%
	MA	SVM + LDA	EEG-fNIRS	Feature-level + Decision-level	92.52%
Meng et al., 2021	MA	LDA	EEG-fNIRS	Feature-level	89.83%
Nour et al., 2021	MI	Convolutional Neural Network (CNN)	EEG-fNIRS	Feature-level	99.85%
**Our work**	MI	Logistic Regression	EEG-fNIRS	Decision-level	96.74%
	MA	Logistic Regression	EEG-fNIRS	Decision-level	98.42%

From the above experimental results, it can be seen that our proposed multimodal fusion method based on multi-level progressive learning with multi-domain features and ASO feature selection is effective and promising in the fusion of EEG-fNIRS. Furthermore, the superiority of this method is verified by comparison with other EEG-fNIRS fusion methods. The proposed method is a novel multimodal fusion algorithm, which combines the advantages of multi-domain feature extraction, the ability of ASO algorithm feature selection and multi-level progressive machine learning, and can effectively fuse the information of EEG and fNIRS to improve the classification accuracy. We validated the effectiveness of the proposed method on two experimental tasks (i.e., MI and MA tasks), and illustrated the superiority of the proposed method through extensive comparisons comparisons with different methods. However, there are still some potential problems in this study that need to be studied and improved. On the one hand, the more multi-domain features extracted from multi-modalities, the richer the brain information contained, and the more comprehensive the task-related intrinsic features. However, as the feature dimension increases, the computation time of the proposed method increases. Therefore, the improvement of the computational efficiency of this method needs to attract more attention in future research. On the other hand, the proposed method was only validated on public datasets and has not been extended to practical BCI applications. Therefore, for future work, we intend to apply the proposed method to solve multimodal BCI classification problems in more real-world situations and to extend the proposed method to a wider range of neuro-clinical applications.

## Conclusion

Aiming at the problems of insufficient feature extraction and insufficient multimodal information fusion in current multimodal fusion, this study proposes a novel multimodal fusion method based on multi-level progressive learning with multi-domain features. The method combines the advantages of multi-domain feature extraction, the feature selection process of ASO algorithm and multi-level progressive machine learning. Based on this method, task-related brain electrical and hemodynamic information can be fully extracted through multi-domain features, the multi-domain features can be eliminated redundant information through ASO algorithm and the multi-domain features of EEG and fNIRS can be effectively fused through multi-level progressive machine learning. The effectiveness and superiority of this method for EEG and fNIRS information fusion are verified on two different tasks, MI and MA. Experiments on the EEG-fNIRS dataset containing 29 subjects show that the proposed method can achieve an average accuracy of 96.74% in the classification of the MI task, and an average accuracy of 98.42% in the classification of the MA task. Our proposed method may provide a general framework for fusion processing of multimodal brain signals and multimodality-based BCI.

## Data availability statement

Publicly available datasets were analyzed in this study. This data can be found here: http://doc.ml.tu-berlin.de/hBCI.

## Ethics statement

The studies involving human participants were reviewed and approved by the Ethics Committee of South China Normal University. The patients/participants provided their written informed consent to participate in this study. Written informed consent was obtained from the individual(s) for the publication of any potentially identifiable images or data included in this article.

## Author contributions

LQ and YZ: conceptualization, resources, and writing-review and editing. LQ, YZ, and ZH: methodology. YZ: software, data curation, and writing-original draft preparation. LQ and JP: validation, supervision, and funding acquisition. LQ, YZ, ZH, and JP: formal analysis and visualization. LQ, ZH, and JP: investigation. LQ: project administration. All authors have read and agreed to the published version of the manuscript.
